# Digital literacy education and problematic Internet use among adolescents: a quasi-experimental study of digital self-efficacy and delay of gratification as cognitive–regulatory correlates

**DOI:** 10.3389/fpsyg.2026.1934152

**Published:** 2026-09-24

**Authors:** Zhaoyu Ding, Yuzhang Fu

**Affiliations:** 1 School of Marxism, Wuhan University, Wuhan, China; 2 Laboratory for Adolescent Values Education, Wuhan University, Wuhan, China

**Keywords:** adolescents, delay of gratification, digital literacy, digital self-efficacy, problematic Internet use, quasi-experimental design

## Abstract

Problematic Internet use (PIU) has become an important concern in adolescent development, yet the psychological correlates through which educational programs may be associated with healthier Internet use remain insufficiently understood. This study examined whether a 6-week multi-component digital literacy and digital wellbeing program was associated with changes in adolescents’ PIU, digital self-efficacy (DSE), and delay of gratification (DGI), and whether the post-intervention data were statistically consistent with a theory-informed pathway linking DSE, DGI, and PIU. A two-arm pretest-posttest quasi-experimental design was conducted with 206 adolescents in Hubei Province, China. Participants were allocated to an intervention group (*n* = 103) or a wait-list control group (*n* = 103) using a quota-based procedure to improve comparability in age, gender, and daily Internet use time. The intervention group received the 6-week program, whereas the wait-list control group was offered the same program after posttest assessment. PIU, DSE, and DGI were measured at pretest and posttest. Data were analyzed using repeated-measures ANOVA and serial mediation analysis with demographic covariates and baseline scores controlled. Significant Group × Time interactions were observed for PIU, DSE, and DGI, indicating more favorable self-reported pre-to-post changes in the intervention group than in the wait-list control group. The post-intervention data were statistically consistent with the hypothesized DSE → DGI → PIU indirect pathway; however, alternative-model analyses indicated that DGI showed the more consistent association with PIU across specifications. These findings provide preliminary quasi-experimental evidence that participation in a multi-component digital literacy and digital wellbeing program is associated with healthier self-reported Internet use, stronger perceived digital competence, and greater delay of gratification. Because participants were not randomly assigned and the mediators and outcome were measured concurrently at posttest, the indirect associations should be regarded as hypothesis-generating rather than as evidence of a causal or temporal sequence.

## 1 Introduction

Problematic Internet use (PIU) has become an important issue in adolescent development because digital media now shape how young people learn, communicate, relax, and construct social identity. Internet use itself is not inherently problematic; for many adolescents, it provides access to information, peer support, and creative opportunities. The concern arises when online engagement becomes difficult to regulate and begins to interfere with sleep, academic participation, emotional adjustment, or offline relationships. A systematic review and meta-analysis found that PIU in the adolescent and young adult populations was related to greater depression, anxiety, and stress levels, suggesting that dysregulated digital engagement is closely linked to psychological distress (Shannon et al., 2022). A separate systematic review further showed that PIU was linked to social anxiety among these populations, indicating that PIU is not only a behavioral issue but also a social–emotional concern (Ding et al., 2023). Evidence from broader adolescent samples has similarly connected Internet addiction with multiple psychological problems, including emotional symptoms and impaired wellbeing (Xue et al., 2023). The issue is particularly salient in China, where adolescents are growing up in one of the world’s largest digital environments. Based on statistics of the 55th Statistical Report on China’s Internet Development, there were approximately 1.108 billion Internet users in China by 2024, and users aged 10–19 accounted for 13.0% of the total Internet population (China Internet Network Information Center, 2025). Recent meta-analytic evidence from Chinese adolescent samples also shows that PIU is significantly associated with suicidal behavior (He et al., 2024a) and non-suicidal self-injury (He et al., 2024b). Collectively, the above results indicate that PIU must be understood not merely as excessive screen time, but as a developmental and public health concern that requires preventive attention.

One promising preventive direction is digital literacy education. Digital literacy is broader than technical skill acquisition. It includes the ability to access, evaluate, interpret, and use digital information responsibly, while also understanding privacy, online risks, platform design, and digital communication. A scoping review has argued that digital literacy is increasingly relevant to health because low digital literacy may limit people’s ability to navigate digital environments and may widen health-related inequalities (Arias López et al., 2023). For adolescents, digital literacy is especially important because online risks are encountered in everyday learning and social contexts rather than in isolated digital episodes. Recent work on youth digital literacy has therefore emphasized not only digital fluency, but also privacy, safety, ethics, empathy, and critical awareness as core components of educational program design (Buchan et al., 2024). In adolescent health contexts, digital and interactive literacy approaches have also been recommended as ways to improve young people’s capacity to make informed decisions in technology-rich environments (Mancone et al., 2024). However, the intervention literature remains uneven. An umbrella review of digital addiction interventions found that existing evidence is promising but heterogeneous, with differences in intervention type, outcome definition, and certainty of evidence across studies (Lu et al., 2025). Similarly, as reported in one systematic review and meta-analysis on health education interventions for adolescents, such programs may reduce Internet addiction and social media addiction outcomes, but also noted variability across intervention designs (Arana-Rodríguez et al., 2026). Individual intervention studies also show that educational programs do not always produce uniform effects. For example, a large school-based program in Japan did not significantly improve Internet or smartphone addiction scores, highlighting the need to understand why some programs work better than others (Otsuka et al., 2023). In contrast, game literacy education has shown potential for improving problematic Internet gaming outcomes, suggesting that targeted literacy content may be useful when it addresses specific digital behaviors (Han et al., 2024). Thus, the field has begun to move beyond the question of whether digital literacy programs change behavior, toward the more precise question of which psychological processes may connect digital literacy education with healthier Internet use.

Digital self-efficacy (DSE) may be one such proximal cognitive process. In the present study, DSE refers to adolescents’ perceived capability to use digital technologies effectively, solve online problems, and manage digital tasks and environments. A digital literacy program may first influence adolescents by strengthening their sense of agency in digital contexts. Instead of experiencing online platforms as environments that simply capture attention, adolescents may begin to feel that they can manage notifications, evaluate content, set boundaries, and make intentional choices. A recent review and scale-development study defined digital self-efficacy as a user’s belief in their ability to perform digital tasks successfully, supporting its role as a confidence-based component of digital competence (Ulfert-Blank and Schmidt, 2022). Empirical work has also shown that Internet self-efficacy is associated with ICT competence and online interaction, indicating that efficacy beliefs are closely related to how young people engage with digital environments (Wu et al., 2023). In the context of PIU, efficacy beliefs are especially relevant because adolescents need not only to know what healthy Internet use means, but also to believe that they can enact it in daily situations. Supporting this interpretation, refusal self-efficacy for Internet use has been found to mediate the association of social support with PIU, suggesting that adolescents who feel capable of resisting inappropriate or excessive Internet use may be less vulnerable to uncontrolled online engagement (Tseng et al., 2024). Therefore, DSE is conceptualized here as a domain-specific cognitive resource that may help adolescents translate digital literacy knowledge into perceived control over digital behavior.

Delay of gratification (DGI) represents a second psychological process, one that is more directly related to self-regulation. Contemporary digital platforms are often built around immediate, frequent, and emotionally salient rewards, including notifications, social feedback, short videos, in-game reinforcement, and algorithmically personalized recommendations. These reward structures make digital engagement attractive, but they also make it difficult for adolescents to postpone immediate online rewards in favor of longer-term goals such as study, sleep, emotional balance, and offline relationships. A meta-analysis found a significant negative association of self-control with Internet addiction among students, demonstrating that weaker self-control is consistently related to higher problematic Internet use (Li et al., 2021). Longitudinal evidence from Chinese adolescents further suggests that Internet self-control and problematic Internet use are reciprocally related over time, which means that self-regulatory difficulties may contribute to PIU while PIU may also weaken later self-control (Wang et al., 2023). Recent adolescent research has also emphasized the key effect of self-regulatory mechanisms on high-risk digital media use, especially when young people must manage competing online and offline demands (Hrbackova et al., 2025). On this basis, DGI is treated in the present study not as a digital behavior itself, but as a broader self-regulatory capacity that may help adolescents resist the immediate rewards embedded in digital platforms.

To establish clear conceptual boundaries, we distinguish the following constructs. Digital literacy refers to the content domain taught in the intervention (e.g., knowledge, critical thinking, and awareness of digital environments). DSE specifically refers to adolescents’ perceived confidence in their ability to use digital tools and solve online problems; it is a cognitive belief, not a measure of actual behavioral self-control. DGI is conceptualized as a generalized regulatory tendency across various life domains (e.g., food, money, social interaction) rather than a digital-specific skill. Finally, self-control represents a broader self-regulatory construct; while DGI is closely related to self-control, we treat them as distinct but overlapping processes in this study.

The present study further proposes that DSE and DGI may be connected in a theory-informed sequence rather than functioning only as separate predictors. A digital literacy program may first strengthen adolescents’ perceived ability to understand and manage digital environments. This enhanced sense of digital competence may then support self-regulatory choices, because adolescents who believe they can control their digital behavior may be more willing and able to postpone immediate online rewards. Prior research on digital self-efficacy supports the idea that perceived competence is an important foundation for adaptive digital engagement (Ulfert-Blank and Schmidt, 2022), while research on refusal self-efficacy suggests that perceived ability to resist problematic Internet use is directly relevant to PIU prevention (Tseng et al., 2024). Meanwhile, evidence on Internet self-control indicates that the ability to regulate online behavior is closely related to lower problematic use (Wang et al., 2023). These findings provide a theoretical basis for the proposed DSE → DGI → PIU sequence. However, this sequence remains a theoretical proposition rather than an established causal mechanism. When mediators and outcomes are measured at the same post-intervention wave, mediation analysis can examine whether the observed data are statistically consistent with the proposed ordering, but it cannot by itself establish definitive temporal or causal mediation.

To address these issues, the present study evaluated a 6-week multi-component digital literacy and digital wellbeing program for adolescents using a two-arm pretest-posttest quasi-experimental design. The first aim was to examine whether adolescents who received the program showed more favorable pre-to-post changes in PIU, DSE, and DGI than adolescents in a wait-list control group. The second aim was to examine whether the post-intervention data were statistically consistent with a theory-informed serial indirect association linking intervention condition, DSE, DGI, and PIU.

Based on prior research and the theoretical rationale outlined above, two hypotheses were proposed. H1: Compared with adolescents in the wait-list control group, adolescents in the digital literacy intervention group would show more favorable pre-to-post changes in PIU, DSE, and DGI, as reflected by significant Time × Group interactions. H2: The association between intervention condition and post-intervention PIU would be statistically consistent with a serial indirect pathway through DSE and DGI, such that intervention condition would be associated with higher DSE, higher DGI, and lower PIU.

## 2 Method

### 2.1 Research design

A two-arm, parallel-group quasi-experimental design with pretest and posttest assessments was employed. The study was conducted in March and April 2026 and was delivered in person at a community center in Hubei Province, China, to ensure standardized implementation of the group-based sessions. The pretest assessment was conducted 1 week prior to the intervention. The posttest assessment was administered within 1 week after the intervention ended (mean interval = 7.0 days; range = 6–8 days), and this interval was similar across all participants. The intervention group received a 6-week digital literacy program during the study period, whereas the wait-list control group did not receive structured digital literacy sessions during this period but was offered the same program after completion of the posttest assessment. Because participants were not randomly assigned to conditions, the study should be interpreted as a quasi-experimental study rather than a randomized controlled trial. Questionnaires were distributed through Wenjuanxing, an online survey platform commonly used in China. Participants completed the questionnaires using anonymous participant codes to protect confidentiality and to match pretest and posttest responses. To maximize retention and reduce missing data, research staff sent standardized reminders through Wenjuanxing and participant communication groups at each assessment wave. Participants completed the questionnaires during dedicated assessment sessions under the online supervision of trained research assistants.

### 2.2 Participants

Participants were 206 adolescents recruited from Hubei Province, China, through online advertisements disseminated via Wenjuanxing and community communication channels. The recruitment notice introduced the study as a digital wellness program for adolescents and included a screening questionnaire. Participants were eligible if they: (a) were aged 12–18 years; (b) had regular Internet access, defined as daily Internet use; (c) had no prior participation in structured digital literacy programs; and (d) had no diagnosed psychiatric disorders as reported by participants or their guardians. To improve comparability on key observed baseline characteristics, participants were allocated using a quota-based procedure after recruitment and baseline assessment. Specifically, a trained research assistant who was not involved in delivering the intervention performed the allocation immediately after all baseline assessments were completed. The allocator was provided only with participants’ age, gender, and baseline daily Internet use time (for quota matching), and was explicitly blinded to their pretest scores on the primary outcome variables (PIU, DSE, and DGI) to minimize selection bias. Although practical factors such as participants’ availability and scheduling preferences were considered to maximize attendance, the final assignment strictly adhered to the pre-defined quota targets to ensure balanced groups across these key characteristics. This allocation procedure was intended to reduce imbalance in observable characteristics between groups; however, because allocation was not randomized, unmeasured confounding cannot be ruled out. To reduce differential expectancy, all participants and guardians were informed that they would receive the digital literacy program either during the study period or after the posttest; thus, the wait-list control group knew they would receive it later but attended no structured sessions beforehand. Regarding assessor blinding, due to the logistical constraints of in-person delivery, research assistants supervising the posttests were aware of group allocation, so blind assessment was not achieved. Furthermore, although participants and guardians knew the program targeted digital wellbeing, they were not explicitly informed of the specific research hypotheses or expected directions of change in the measured variables (e.g., DSE, DGI, or PIU).

A sensitivity power analysis was conducted to evaluate whether the available sample size was adequate for detecting the primary Group × Time interaction. With 206 participants, two groups, two repeated measurements, *α* = 0.05, and statistical power of 0.80, the study had sufficient power to detect a small-to-medium interaction effect. Therefore, the sample size was considered acceptable for the primary repeated-measures analysis. Nevertheless, the mediation analysis should be interpreted cautiously because the study was not originally powered specifically for detecting indirect effects.

### 2.3 Intervention: multi-component digital literacy and digital wellbeing program

The intervention was a multi-component digital literacy and digital wellbeing program. It consisted of 6 weekly group sessions, each lasting approximately 45–60 min. The program was delivered in person at the community center and combined scenario-based instruction, guided discussion, interactive activities, and behavioral practice. The curriculum was designed not only to provide information about Internet risks, but also to help adolescents develop practical skills for understanding digital environments, regulating online behavior, managing emotions related to Internet use, and maintaining a healthier balance between online and offline activities. While the program includes digital literacy content (e.g., platform awareness, privacy, citizenship), it also explicitly and directly targets broader self-regulatory skills (e.g., self-control and delay of gratification in Session 3, and time management in Session 5). The Interaction of Person–Affect–Cognition–Execution (I-PACE) model was used to inform the overall intervention logic. It conceptualizes problematic Internet-related behaviors as involving interactions across individual features, affectional and cognitive responses, and executive control (Brand et al., 2019). This model was used specifically to guide sessions addressing PIU awareness, platform reward mechanisms, and executive control in Internet use. The program also drew on evidence that self-control is negatively associated with Internet addiction among students, which supported the inclusion of self-control and delay-of-gratification training in Session 3 (Li et al., 2021). Longitudinal evidence showing reciprocal relations of Internet self-control with PIU in adolescents in China further supported the emphasis on Internet-specific self-regulation and time-management practice in Session 5 (Wang et al., 2023). The session on emotional Internet use was guided by emotion regulation theory, with emotion modulation being defined as processes where individuals affect their emotion type, emotion occurrence timing, and emotion experience and expression ways (Gross, 2015). The program’s broader digital wellbeing orientation was informed by recent evidence distinguishing digital competency from digital dependency, suggesting that healthy digital functioning requires both competence in digital environments and reduced dependency-related difficulties (Chen et al., 2025). Finally, the curriculum was consistent with recent adolescent research showing that self-control, sense of control, and digital media self-regulation are associated with lower risky digital media use, including excessive and impulsive engagement (Hrbackova et al., 2025). Each session followed a three-part structure. First, facilitators introduced the session topic through age-appropriate examples and digital-use scenarios. Second, participants completed interactive activities, such as reflecting on their own Internet-use habits, identifying platform reward mechanisms, discussing emotional triggers for excessive use, and practicing time-management strategies. Third, participants were assigned post-session practice tasks, including daily time-planning exercises, goal setting, and behavioral challenges such as completing 1 h of phone-free time per day. These tasks were intended to help participants transfer the session content into everyday digital behavior. All sessions were delivered by trained facilitators following a standardized curriculum outline and activity checklist. This standardized format was used to improve consistency across group sessions. The wait-list control group received no structured digital literacy sessions during the study period but were informed that they would receive the same program after completion of the posttest assessment. The full digital literacy program curriculum, including session plans, activity guides, and post-class practice assignments, was originally developed in Chinese and can be obtained on request from the corresponding author. An English summary of program content is provided in Table 1.

**Table 1:** Digital literacy program curriculum structure.

Session	Theme	Core objectives	Theoretical or empirical basis
Session 1	Digital World and Online Behavior	Introduce common Internet functions and platform features; guide adolescents to reflect on their personal Internet-use habits and recognize patterns of unplanned or excessive use.	Digital literacy and digital wellbeing perspective
Session 2	Problematic Internet Use and Platform Reward Mechanisms	Help participants identify signs of PIU; explain how notifications, recommendation algorithms, social feedback, and game-like rewards may reinforce repeated online engagement.	I-PACE model (Brand et al., 2019)
Session 3	Self-Control and Delay of Gratification	Discuss immediate versus delayed rewards using age-appropriate examples, including the marshmallow task; practice strategies for postponing online gratification and resisting impulsive checking.	Self-control and Internet addiction evidence (Li et al., 2021); delay-of-gratification perspective
Session 4	Emotion Management and Internet Use	Identify emotional triggers for excessive Internet use; discuss emotional Internet use as a coping strategy; practice alternative coping and emotion regulation strategies.	Emotion regulation theory (Gross, 2015)
Session 5	Healthy Internet Use and Time Management	Develop time-planning strategies; balance study, entertainment, sleep, and offline activities; practice attention and concentration strategies.	Internet self-control and PIU evidence (Wang et al., 2023); I-PACE executive control component
Session 6	Digital Citizenship and Real-World Connection	Discuss online etiquette, cyberbullying awareness, privacy protection, and responsible communication; emphasize offline relationships and real-world social participation.	Digital citizenship and digital wellbeing perspective

PIU = problematic Internet use; I-PACE = Interaction of Person–Affect–Cognition–Execution. The table provides an English summary of the Chinese intervention curriculum. The full curriculum materials can be obtained from the corresponding author on request.

To support intervention fidelity, facilitators received standardized training before program delivery and used a session checklist for each weekly session. The checklist covered the core topic, scenario-based instruction, interactive activity, discussion component, and post-session practice assignment. Research staff reviewed these checklists after each session to ensure that the main curriculum components had been delivered consistently. Although no independent external fidelity rating was conducted, these procedures were used to improve implementation consistency. Attendance was recorded for all participants in the intervention group: on average, participants attended 5.64 out of 6 sessions (SD = 0.74), with 69.9% (*n* = 72) attending all six sessions, 24.3% (*n* = 25) attending five sessions, and only 5.8% (*n* = 6) attending four sessions. Participants who missed a session received a brief make-up summary and the corresponding practice task. To examine whether intervention effects were influenced by differential exposure, we conducted an exploratory per-protocol (PP) analysis, comparing participants who attended all six sessions (*n* = 72) with the wait-list control group (*n* = 103).

### 2.4 Measures

The instruments were administered in Chinese. For scales that did not have an established Chinese adolescent validation in the exact form used in this study, a standardized translation and cultural adaptation procedure was conducted before data collection. All English items were first translated into Chinese by two independent bilingual researchers. A third bilingual researcher was later invited to back-translate the Chinese version into English. Discrepancies between the translated and back-translated versions were discussed by the research team and resolved by consensus. Three experts in adolescent psychology, educational psychology, and digital behavior research reviewed the translated items for semantic equivalence, developmental appropriateness, and cultural relevance. A pilot test was then conducted with 20 adolescents not recruited into our final sample. Minor wording was adjusted based on participants’ feedback to improve clarity and age-appropriate comprehension. The R lavaan package (Rosseel, 2012) was employed for confirmatory factor analysis (CFA). Maximum likelihood estimation with robust standard errors was employed for model estimation. The comparative fit index (CFI), Tucker–Lewis index (TLI), standardized root mean square residual (SRMR), and root mean square error of approximation (RMSEA) were adopted for evaluating model fit. CFI and TLI values ≥ 0.90 and RMSEA and SRMR values ≤ 0.08 were deemed to be acceptable. The chi-square to degrees of freedom ratio (*χ*^2^/df) was expressed as a supplementary descriptive index, with values below 3 indicating acceptable fit.

#### 2.4.1 Problematic Internet use

Problematic Internet use was measured using the Short Problematic Internet Use Test (SPIUT; Siciliano et al., 2015). The SPIUT is a six-item instrument developed to assess problematic Internet use among adolescents. Items are rated on a five-point Likert scale, with higher scores indicating higher levels of problematic Internet use. All six items are positively worded, and no reverse-coded items are included. A sample item is: “Do you find that you are staying online longer than you intended?” The scale exhibited high internal consistency within this sample, and Cronbach’s *α* was 0.82 at pretest and 0.85 at posttest. According to CFA, the SPIUT had a one-factor structure within this sample: *χ*^2^/df = 1.689, CFI = 0.992, TLI = 0.987, RMSEA = 0.058 [90% CI: 0.000, 0.103], and SRMR = 0.030.

#### 2.4.2 Digital self-efficacy

The Internet Self-Efficacy subscale of the Adolescent Internet Adaptability Scale (AIAS; Wang et al., 2025) was employed for assessing digital self-efficacy. The AIAS was developed for assessing adaptive abilities of adolescents in Internet use and includes eight dimensions, one of which is Internet self-efficacy. The Internet Self-Efficacy subscale contains five items assessing adolescents’ perceived competence in using the Internet effectively and managing online tasks. Items are rated by a six-point scale (with 1 = not like me at all, and 6 = exactly like me), with higher scores indicating stronger digital self-efficacy. All items are positively worded, and no reverse-coded items are included. A sample item is: “I am confident in my ability to create my own content on the Internet.” The five-item subscale exhibited acceptable internal consistency within this study, with Cronbach’s *α* being 0.79 at pretest and 0.83 at posttest. CFA supported a one-factor structure: *χ*^2^/df = 1.096, CFI = 0.999, TLI = 0.998, RMSEA = 0.022 [90% CI: 0.000, 0.100], and SRMR = 0.017.

#### 2.4.3 Delay of gratification

Delay of gratification was measured using the short-form Delaying Gratification Inventory (DGI; Hoerger et al., 2011). This short form includes 10 items covering five content dimensions: food, physical pleasure, social interaction, money, and achievement. The five-point Likert scale was employed for rating items, and higher scores indicate stronger delay-of-gratification capacity. In this scale, positive and negative wording items are included, with negative wording items being reverse-scored before analysis. A sample reverse-scored item is: “I would have a hard time sticking with a special, healthy diet.” Although the DGI includes multiple content domains, the total score was used in the present study as an indicator of general delay-of-gratification capacity. However, we acknowledge that the DGI items are not specific to digital behavior, capturing generalized self-regulation rather than digital-specific delay. The scale showed acceptable internal consistency, with Cronbach’s *α* = 0.76 at pretest and 0.81 at posttest. CFA indicated acceptable model fit in this sample: *χ*^2^/df = 1.857, CFI = 0.992, TLI = 0.990, RMSEA = 0.065 [90% CI: 0.040, 0.088], and SRMR = 0.023.

Regarding behavioral outcomes, although daily Internet use time was assessed at baseline, objective behavioral measures (e.g., app-use logs or device screen-time records) were not collected at posttest due to the logistical constraints of the anonymous online survey platform used in this community-based study. Consequently, the primary outcomes were based solely on self-report. This limitation is further addressed in the Discussion section.

### 2.5 Data analysis

SPSS 27.0 and the PROCESS macro for SPSS were employed for data analysis. Before the main analyses, missing data, univariate outliers, and distributional characteristics were examined. Univariate outliers on continuous variables were examined using *z*-scores and boxplots. Demographics and study data were expressed as descriptive statistics. Independent-samples *t*-tests (for continuous data) and chi-square tests (categorical data) were employed to examine baseline equivalence between two groups. The primary intervention analysis was conducted using 2 × 2 repeated-measures ANOVA, where group was a between-subjects factor whereas time was a within-subjects factor. The Group × Time interaction was treated as the primary test of whether pre-to-post changes differed between two groups. We expressed partial eta squared (*ηp*^2^) as the effect size for ANOVA. Paired-samples *t*-tests were then used as descriptive follow-up analyses to examine within-group pre-to-post changes, while independent-samples *t*-tests were utilized for comparing posttest scores in two groups. Cohen’s *dz* was reported for paired-samples comparisons, while Cohen’s *d* was reported for independent-samples analyses. *α* = 0.05 indicated significant differences (two-sided). To examine whether post-intervention data were statistically consistent with the proposed serial pathway, Hayes’ PROCESS macro, Model 6 was adopted for testing the mediation model (Hayes, 2017). Intervention condition was entered as the independent variable, with the wait-list control and intervention groups coded as 0 and 1 separately. Post-intervention digital self-efficacy was entered as the first mediator, post-intervention delay of gratification as the second mediator, and post-intervention problematic Internet use as the dependent variable. Gender, age, daily Internet use time, and pretest scores of digital self-efficacy, delay of gratification, and problematic Internet use were included as covariates. Bias-corrected bootstrap confidence intervals were used to test indirect effects based on 5,000 resamples. Indirect effects were considered statistically significant when the 95% confidence interval did not include zero. Because the mediators and outcome were measured at the same post-intervention wave, the mediation model was interpreted as a test of statistical consistency with the proposed theoretical pathway rather than as definitive evidence of temporal or causal mediation. Because the dispersion of DGI scores differed markedly across groups and waves, the primary DGI interaction was supplemented by a Welch independent-samples *t* test comparing individual change scores (posttest minus pretest) between groups. For the exploratory per-protocol analysis, posttest scores were regressed on full-attendance status, the corresponding pretest score, and baseline daily Internet-use duration; heteroscedasticity-consistent HC3 standard errors were used. These supplementary analyses were treated as robustness checks and did not replace the primary assigned-group analysis.

## 3 Results

### 3.1 Participant flow and basic features

A total of 246 adolescents were assessed for eligibility through online screening. Of these, 27 did not meet the inclusion criteria, 9 declined to participate before baseline assessment, and 4 could not be contacted. The remaining 206 eligible participants completed the pretest assessment and were allocated into intervention (*n* = 103) and wait-list control (*n* = 103) groups using a quota-based procedure. All 206 participants completed the posttest assessment and were enrolled for final analysis, yielding a 100% retention rate. This complete retention was likely facilitated by the short study period, dedicated assessment sessions, and standardized reminders at each assessment wave. Figure 1 presents the participant flow.

**Figure 1:**
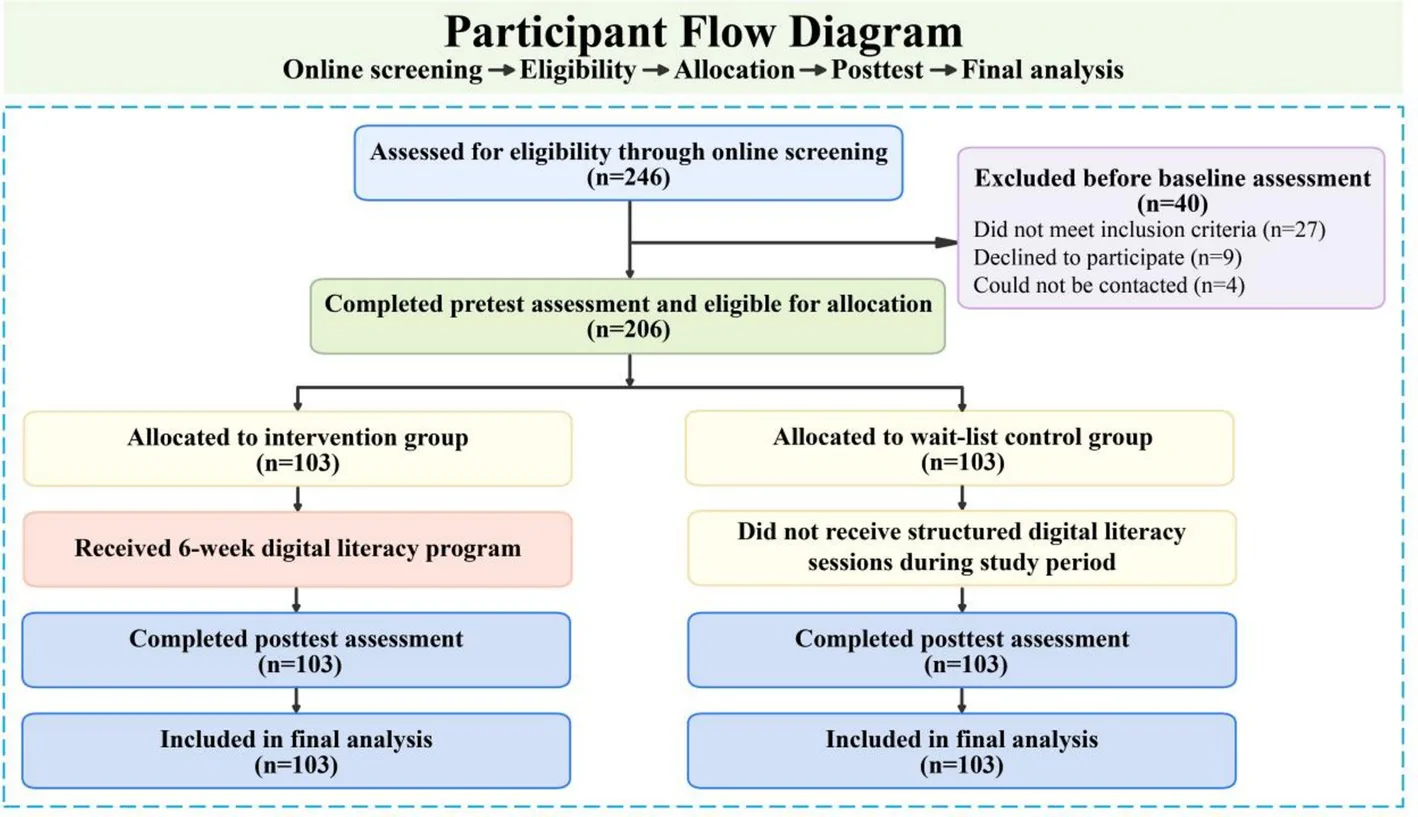
Participant flowchart.

Table 2 presents the basic demographics in two groups. Gender distribution, age, and daily Internet use time were not significantly different between two groups (all *p* > 0.05). In addition, independent-samples *t*-tests showed no statistically significant baseline differences between the two groups in PIU, DGI, or DSE at pretest (all *p* > 0.05; see Table 3). These results indicated that the two groups were comparable on the observed baseline characteristics included in the quota-based allocation procedure.

**Table 2:** Baseline demographic characteristics by group.

Variable	Intervention group (*n* = 103)	Wait-list control group (*n* = 103)	Test statistic	*p*
Gender, *n* (%)			*χ*^2^ = 0.31	0.578
Male	50 (48.5%)	54 (52.4%)		
Female	53 (51.5%)	49 (47.6%)		
Age (years), M (SD)	13.98 (1.50)	13.83 (1.41)	*t* = 0.74	0.461
Daily Internet use, *n* (%)			*χ*^2^ = 0.93	0.920
<1 h	11 (10.7%)	13 (12.6%)		
1–2 h	29 (28.2%)	27 (26.2%)		
2–3 h	29 (28.2%)	26 (25.2%)		
3–6 h	23 (22.3%)	25 (24.3%)		
>6 h	11 (10.7%)	12 (11.7%)		

Percentages are calculated within each group.

**Table 3:** Descriptive statistics and within-group changes by group.

Outcome	Group	Pretest M ± SD	Posttest M ± SD	Within- group *t*	*p*	Cohen’s *dz*
PIU	Intervention	19.35 ± 5.76	13.50 ± 4.67	10.20	<0.001	1.01
PIU	Wait-list control	19.65 ± 4.74	17.08 ± 5.55	3.84	<0.001	0.38
DGI	Intervention	30.14 ± 2.16	38.04 ± 6.62	−12.44	<0.001	1.23
DGI	Wait-list control	30.12 ± 2.23	31.58 ± 10.68	−1.36	0.176	0.13
DSE	Intervention	19.18 ± 6.52	23.26 ± 4.93	−8.15	<0.001	0.80
DSE	Wait-list control	19.03 ± 6.10	18.62 ± 5.95	0.45	0.655	0.04

PIU, problematic Internet use; DGI, delay of gratification; DSE, digital self-efficacy. Cohen’s *dz* was reported for paired-samples comparisons.

### 3.2 Group differences in pre-to-post changes

Group differences in pre-to-post changes were examined using 2 × 2 repeated-measures ANOVA, with group as the between-subjects factor and time as the within-subjects factor. The Group × Time interaction was treated as the primary test of whether changes differed between the intervention and wait-list control groups. As shown in Table 4, significant Group × Time interactions were observed for PIU, *F*(1, 204) = 13.75, *p* < 0.001, *ηp*^2^ = 0.063; DGI, *F*(1, 204) = 26.53, *p* < 0.001, *ηp*^2^ = 0.115; and DSE, *F*(1, 204) = 18.62, *p* < 0.001, ηp^2^ = 0.084. These findings indicated that the magnitude of pre-to-post change differed significantly between the two groups. Descriptive follow-up analyses showed that PIU decreased in both groups, but the reduction was larger in the intervention group. In the intervention group, PIU decreased from 19.35 ± 5.76 at pretest to 13.50 ± 4.67 at posttest, *t*(102) = 10.20, *p* < 0.001, Cohen’s *dz* = 1.01. In the wait-list control group, PIU also decreased from 19.65 ± 4.74 to 17.08 ± 5.55, *t*(102) = 3.84, *p* < 0.001, Cohen’s *dz* = 0.38. For DGI, the intervention group showed a significant increase from 30.14 ± 2.16 to 38.04 ± 6.62, *t*(102) = −12.44, *p* < 0.001, Cohen’s *dz* = 1.23, whereas the change in the wait-list control group was not statistically significant, from 30.12 ± 2.23 to 31.58 ± 10.68, *t*(102) = −1.36, *p* = 0.176, Cohen’s *dz* = 0.13. For DSE, the intervention group showed a significant increase from 19.18 ± 6.52 to 23.26 ± 4.93, *t*(102) = −8.15, *p* < 0.001, Cohen’s *dz* = 0.80, whereas the wait-list control group showed no significant change, from 19.03 ± 6.10 to 18.62 ± 5.95, *t*(102) = 0.45, *p* = 0.655, Cohen’s *dz* = 0.04. Posttest comparisons further showed significant group differences in PIU, DGI, and DSE, with the intervention group reporting lower PIU and higher DGI and DSE than the wait-list control group. Overall, the significant Group × Time interactions, rather than within-group changes alone, were regarded as the primary evidence of differential change between groups.

**Table 4:** Results of 2 × 2 repeated-measures ANOVA.

Outcome	Source	df	*F*	*p*	*ηp* ^2^
PIU	Time	1,204	91.04	<0.001	0.309
	Group	1,204	11.34	<0.001	0.053
	Time × Group	1,204	13.75	<0.001	0.063
DGI	Time	1,204	56.21	<0.001	0.216
	Group	1,204	24.81	<0.001	0.108
	Time × Group	1,204	26.53	<0.001	0.115
DSE	Time	1,204	12.47	<0.001	0.058
	Group	1,204	14.14	<0.001	0.065
	Time × Group	1,204	18.62	<0.001	0.084

*ηp*^2^ = partial eta squared.

The significant Group × Time interactions indicated that the magnitude of pre-to-post change differed between the intervention and wait-list control groups. Follow-up analyses showed that PIU decreased in both groups, but the reduction was larger in the intervention group. DGI and DSE increased significantly in the intervention group, whereas changes in the wait-list control group were not statistically significant. Therefore, the Group × Time interactions, rather than within-group changes alone, were treated as the primary evidence of differential change.

As a supplementary analysis, participants who attended all six sessions (*n* = 72) were compared with the wait-list control group (*n* = 103) using baseline-adjusted linear models with HC3 robust standard errors. Models adjusted for the corresponding pretest outcome and baseline daily Internet-use duration. Full attendance was associated with lower posttest PIU (*B* = −3.95, SE = 0.76, 95% CI [−5.44, −2.46], *p* < 0.001), higher posttest DGI (*B* = 7.18, SE = 1.33, 95% CI [4.56, 9.80], *p* < 0.001), and higher posttest DSE (*B* = 4.62, SE = 0.85, 95% CI [2.94, 6.29], *p* < 0.001). Because attendance was not randomly determined, these estimates are descriptive and may reflect motivation, family support, or other adherence-related differences; they complement but do not replace the primary assigned-group findings.

Because DGI dispersion increased markedly from pretest to posttest, particularly in the wait-list group, item coding, reverse scoring, participant linkage across waves, missing-data handling, and total-score calculations were rechecked. No missing values, duplicate participant codes, or scores outside the theoretical range were identified. In the intervention group, the pretest median was 30.00 (Q1 = 29.00, Q3 = 31.50; range = 25–35) and the posttest median was 39.00 (Q1 = 32.00, Q3 = 42.50; range = 22–50). In the wait-list group, the pretest median was 30.00 (Q1 = 29.00, Q3 = 32.00; range = 24–36) and the posttest median was 31.00 (Q1 = 21.00, Q3 = 41.00; range = 11–48). Boxplot inspection identified two mild pretest outliers, one in each group (scores of 25 and 24, respectively), and no posttest values beyond the group-specific 1.5 × IQR fences. The marked wait-list posttest dispersion was therefore retained as an observed feature of the data rather than attributed to a scoring error; nevertheless, its source cannot be determined from the available data and the DGI findings warrant corresponding caution.

To test the differential change directly without assuming equal group variances, individual DGI change scores were compared using Welch’s *t* test. Mean change was 7.90 points in the intervention group and 1.47 points in the wait-list group, a between-group difference of 6.44 points, *t*(165.41) = 5.15, *p* < 0.001, Cohen’s *d* = 0.72. A rank-based comparison of change scores yielded the same conclusion (Mann–Whitney *U* = 7,059.50, *p* < 0.001). Thus, the evidence for differential DGI change was retained under analyses less sensitive to unequal variances, although the unusual increase in wait-list dispersion remains a limitation.

### 3.3 Assumption checks and correlations

Because all primary variables were evaluated by self-report measures, it was impossible to fully eliminate common-method bias. As a descriptive check, we used Harman’s single-factor test with the unrotated exploratory factor analysis of post-intervention self-report variables. The first unrotated factor explained 33.16% of the total variance. This result was used only as a descriptive diagnostic and should not be interpreted as definitive evidence that common-method bias was absent. Skewness values were between −0.552 and 0.404, whereas kurtosis values were between −2.020 and −0.046, suggesting absence of serious deviation from normality. VIF values were 1.016–4.839, while tolerance values were 0.207–0.985. The Durbin–Watson statistic was 1.909. These values did not indicate serious multicollinearity or autocorrelation. Table 5 shows means, standard deviations, alongside Pearson correlation coefficients of primary post-intervention variables. Group was coded as 0 = wait-list control group and 1 = intervention group. Group was positively correlated with DSEpost and DGIpost and negatively related to PIUpost. DSEpost was positively related to DGIpost but negatively related to PIUpost. DGIpost was also negatively correlated with PIUpost. These correlations provided preliminary support for testing the proposed mediation model, while not establishing causal or temporal ordering across all variables.

**Table 5:** Means, standard deviations, and correlations among variables (*N* = 206).

Variable	Mean	SD	1	2	3	4
1. Group (0 = wait-list control, 1 = intervention)	0.50	0.50	1			
2. DSEpost	20.94	5.93	0.392^***^	1		
3. DGIpost	34.81	9.43	0.343^***^	0.784^***^	1	
4. PIUpost	15.29	5.42	−0.330^***^	−0.564^***^	−0.762^***^	1

*N* = 206. Group was coded as 0 = wait-list control group and 1 = intervention group. DSEpost = post-intervention digital self-efficacy; DGIpost = post-intervention delay of gratification; PIUpost = post-intervention problematic Internet use. ^***^p < 0.001.

### 3.4 Serial mediation analysis

A serial mediation model was carried out with PROCESS Model 6 by adopting 5,000 bootstrap samples for examining if post-intervention data were statistically consistent with the proposed cognitive–regulatory pathway. Intervention condition represented an independent variable, with the wait-list control and intervention groups being coded as 0 and 1 separately. DSEpost and DGIpost were entered as the first and second mediators, respectively, and PIUpost was entered as the dependent variable. Gender, age, daily Internet use time, and pretest scores of DSE, DGI, and PIU were included as covariates. Table 6 presents the regression results with unstandardized coefficients. Intervention condition was positively associated with DSEpost, *B* = 4.784, SE = 0.781, *p* < 0.001. DSEpost was positively associated with DGIpost, *B* = 1.307, SE = 0.073, *p* < 0.001, whereas the direct association between intervention condition and DGIpost was not statistically significant, *B* = 0.132, SE = 0.886, *p* = 0.882. In the final model predicting PIUpost, DGIpost was negatively associated with PIUpost, *B* = −0.414, SE = 0.042, *p* < 0.001, whereas the direct associations of intervention condition and DSEpost with PIUpost were not statistically significant. The final model explained 64.3% of the variance in PIUpost.

**Table 6:** Sequential mediation regression results with covariates included.

Outcome	Predictor	*R* ^2^	*F*	*B*	SE	*t*	*p*
DSEpost	Group	0.208	8.697^***^	4.784	0.781	6.129	<0.001
DGIpost	Group	0.666	56.385^***^	0.132	0.886	0.149	0.882
	DSEpost			1.307	0.073	17.797	<0.001
PIUpost	Group	0.643	44.423^***^	−1.024	0.525	−1.951	0.052
	DSEpost			−0.012	0.073	−0.165	0.869
	DGIpost			−0.414	0.042	−9.790	<0.001

Covariates included gender, age, daily Internet use time, DSEpre, DGIpre, and PIUpre. Group was coded as 0 = wait-list control group and 1 = intervention group. DSEpost = post-intervention digital self-efficacy; DGIpost = post-intervention delay of gratification; PIUpost = post-intervention problematic Internet use; *B* = unstandardized coefficient; SE = standard error. ***p < 0.001 (for the overall model F test).

Table 7 presents the total, direct, and indirect effects. The total effect of intervention condition on PIUpost was significant, *B* = −3.724, 95% CI [−5.155, −2.292], whereas the direct effect was not statistically significant, *B* = −1.024, 95% CI [−2.059, 0.011]. The total indirect effect was significant, *B* = −2.700, 95% CI [−3.984, −1.571]. Among the three specific indirect paths, only the sequential path from intervention condition to DSEpost, then to DGIpost, and finally to PIUpost was significant, *B* = −2.589, 95% CI [−3.729, −1.675]. The two single-mediator paths showed no significance since zero was involved in their CIs.

**Table 7:** Total, direct, and indirect effects of group on PIUpost via DSEpost and DGIpost (covariates included).

Path	*B*	BootSE	95% BootCI
Total effect (group → PIUpost)	−3.724	0.726	[−5.155, −2.292]
Direct effect (group → PIUpost)	−1.024	0.525	[−2.059, 0.011]
Total indirect effect	−2.700	0.608	[−3.984, −1.571]
Ind1: group → DSEpost → PIUpost	−0.057	0.367	[−0.768, 0.682]
Ind2: group → DGIpost → PIUpost	−0.055	0.350	[−0.733, 0.650]
Ind3: group → DSEpost → DGIpost → PIUpost	−2.589	0.527	[−3.729, −1.675]

Covariates included gender, age, daily Internet use time, DSEpre, DGIpre, and PIUpre. Group was coded as 0 = wait-list control group and 1 = intervention group. BootSE = bootstrap standard error; BootCI = bootstrap confidence interval.

To facilitate interpretation, Figure 2 presents the standardized serial mediation model. Table 7 displays the unstandardized indirect effects, whereas Figure 2 displays standardized path coefficients. Intervention condition had positive relation with DSEpost, *β* = 0.404, 95% CI [0.273, 0.534]. DSEpost was positively associated with DGIpost, *β* = 0.821, 95% CI [0.731, 0.913]. DGIpost showed negative association with PIUpost, *β* = −0.721, 95% CI [−0.865, −0.576]. The standardized serial indirect effect was significant, *β* = −0.478, 95% CI [−0.664, −0.313], whereas those direct paths from intervention condition to DGIpost, from DSEpost to PIUpost, and from intervention condition to PIUpost were not statistically significant. Taken together, the mediation results were statistically consistent with the proposed serial pathway. Intervention condition was associated with higher DSEpost, which was associated with higher DGIpost, which in turn was associated with lower PIUpost. However, because the mediators and outcome were measured at the same post-intervention wave, this model should be interpreted as evidence of statistical consistency with the proposed theoretical pathway rather than as definitive evidence of temporal or causal mediation.

**Figure 2:**
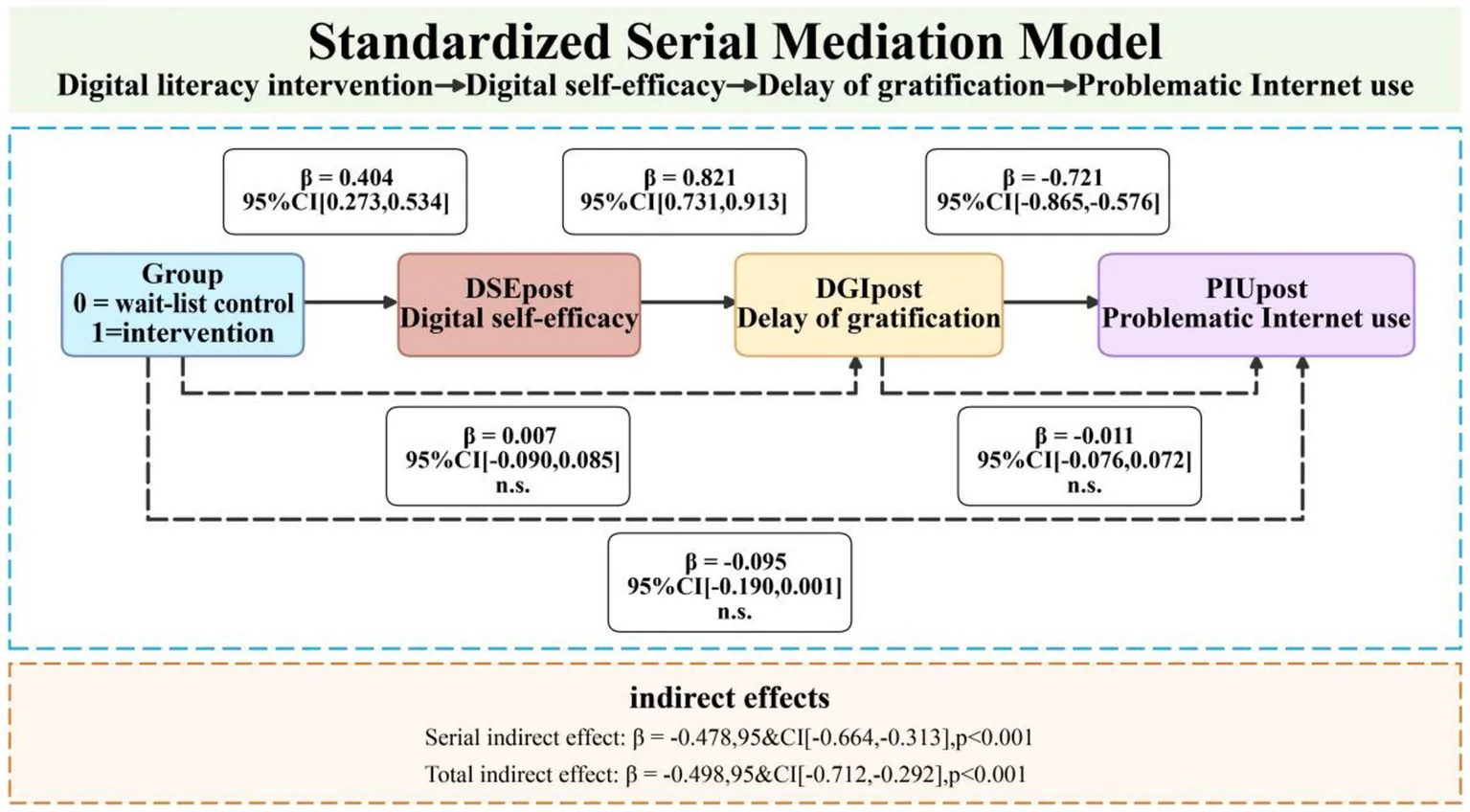
Standardized serial mediation model linking intervention condition, digital self-efficacy, delay of gratification, and problematic Internet use. Values are standardized coefficients with bootstrap 95% confidence intervals. The model controlled for gender, age, daily Internet use time, and pretest scores of PIU, DSE, and DGI. Group was coded as 0 = wait-list control group and 1 = intervention group. Solid arrows indicate statistically significant paths, whereas dashed arrows indicate non-significant paths. DSEpost = post-intervention digital self-efficacy; DGIpost = post-intervention delay of gratification; PIUpost = post-intervention problematic Internet use. Because DSE, DGI, and PIU were measured concurrently at posttest, the model should be interpreted as statistical consistency with the proposed pathway rather than evidence of temporal mediation.

Given the high intercorrelation between DSEpost and DGIpost (*r* = 0.784) and their concurrent measurement, two supplementary models were examined. In a reverse serial model (Group → DGIpost → DSEpost → PIUpost), the sequential indirect association was not statistically significant (*B* = −0.063, 95% CI [−0.559, 0.407]). In a parallel-mediator model, the specific indirect association through DGIpost was significant (*B* = −2.688, 95% CI [−3.940, −1.615]), whereas the specific indirect association through DSEpost was not (*B* = −0.096, 95% CI [−0.818, 0.599]). These alternative specifications do not establish temporal priority between DSE and DGI. Rather, they show that the substantive interpretation depends on model specification and that DGI had the more consistent association with PIU across the examined models. The hypothesized DSE → DGI ordering should therefore be regarded as one theory-informed representation that requires longitudinal verification.

## 4 Discussion

### 4.1 Principal findings

This quasi-experimental study examined whether a 6-week multi-component digital literacy and digital wellbeing program was associated with changes in adolescents’ problematic Internet use, digital self-efficacy, and delay of gratification. Significant Group × Time interactions were observed for all three outcomes, indicating that adolescents in the intervention group showed more favorable pre-to-post changes than those in the wait-list control group. Specifically, the intervention group reported lower PIU and higher DSE and DGI after the program. These findings suggest that a structured digital literacy program may be a useful educational approach for supporting healthier Internet use among adolescents. At the same time, the findings should be interpreted with appropriate caution. PIU also decreased significantly in the wait-list control group, regardless of the lower change magnitude than the intervention group. Such pattern may reflect assessment participation, expectancy, increased self-monitoring, or natural fluctuation during the study period. Therefore, the significant Group × Time interactions, rather than within-group changes alone, should be regarded as the primary evidence of differential change between groups. The mediation results showed that the post-intervention data were statistically consistent with the proposed cognitive-regulatory pathway, while alternative specifications indicated that DGI had the more consistent association with PIU. Intervention condition was related to greater post-intervention DSE, which was linked to greater post-intervention DGI, which in turn was linked to smaller post-intervention PIU. Notably, the sequential indirect path through DSE and DGI was significant, whereas the single-mediator paths through DSE alone and DGI alone were not. This pattern suggests that DSE and DGI may be more informative when considered as linked processes rather than as isolated predictors. However, because DSE, DGI, and PIU were measured at the same post-intervention wave, the mediation findings should be understood as evidence of statistical consistency with a theory-informed pathway, not as definitive evidence that changes in DSE temporally preceded changes in DGI or that DGI causally reduced PIU.

### 4.2 Theoretical contributions

The present results help theoretically understand how digital literacy education may relate to adolescent PIU. Rather than treating digital literacy as a purely knowledge-based intervention, the results suggest that adolescents’ perceived competence in digital environments and their self-regulatory capacity may be important psychological correlates of healthier Internet use. This interpretation conforms to social-cognitive theory, which emphasizes that self-efficacy beliefs are important for goal-directed behavior and self-regulation (Bandura, 1986). In the present study, DSE appeared as the first variable in the statistically supported indirect pathway, suggesting that adolescents who felt more capable of managing digital environments also tended to report stronger delay-of-gratification capacity. The findings are also compatible with the I-PACE model, which emphasizes the interaction of individual characteristics, affective and cognitive responses, and executive control in problematic Internet-related behaviors (Brand et al., 2019). From this perspective, digital self-efficacy may reflect a cognitive resource that helps adolescents approach online environments with a stronger sense of control, whereas delay of gratification may reflect a regulatory capacity that helps them resist immediate online rewards. The observed DSE → DGI → PIU pattern therefore provides preliminary support for linking cognitive empowerment with self-regulation in adolescent digital behavior. Importantly, the present findings do not prove that digital self-efficacy and delay of gratification operate in a fixed causal order. They do, however, suggest that the benefits of the intervention may extend beyond the simple transmission of risk-related knowledge. Because digital literacy knowledge was not directly measured in this study, we cannot determine whether knowledge changed or whether such change contributed to the outcomes. Instead, the present findings highlight that perceived digital competence (DSE) and self-regulatory capacity (DGI) may represent additional, complementary pathways through which such programs may relate to healthier Internet use. Given that the intervention included direct training on these skills (Sessions 3 and 5), the observed changes in DGI may reflect both direct instructional effects and the hypothesized indirect effect through DSE. Social Cognitive Theory provides a rationale for considering DSE before self-regulatory behavior, but the present concurrent data cannot determine whether DSE preceded DGI, whether DGI preceded DSE, or whether they reflected overlapping processes. This provides a useful direction for future research: digital literacy programs may be better understood not only by asking whether they reduce problematic use, but also by examining how they shape adolescents’ confidence, self-control, and everyday decision-making in digital environments.

### 4.3 Implications for digital literacy education

These results have some implications for designing adolescent digital literacy programs. First, digital literacy education may benefit from moving beyond one-time informational sessions about the risks of excessive Internet use. Adolescents often already know that uncontrolled Internet use can interfere with study, sleep, and relationships. What may be more difficult is translating this knowledge into daily choices when they are faced with notifications, social feedback, short videos, games, and other immediate digital rewards. Programs that combine knowledge, reflection, practice, and behavioral challenges may therefore be more useful than programs that rely only on warning-based instruction. Second, the present findings suggest a potentially useful hypothesis for future research: that digital self-efficacy-building activities may be appropriately placed early in the curriculum. Activities such as identifying personal Internet-use patterns, learning how platform features influence attention, managing notifications, setting boundaries, and practicing digital problem-solving may help adolescents develop a stronger sense of agency. Once adolescents believe that they can manage their digital environment, they may be better prepared to engage in more demanding self-regulatory practices. However, because this study did not experimentally manipulate the sequence of curriculum components, this specific ordering remains a testable hypothesis rather than an established instructional principle. Future research should directly compare different program designs (e.g., competence-building first vs. self-control training first) to examine this possibility. Third, delay-of-gratification training may serve as an important bridge between digital competence and healthier Internet behavior. In the present program, activities such as discussing immediate versus delayed rewards, adapting the marshmallow task to digital scenarios, setting daily goals, and practicing phone-free time were intended to help adolescents experience self-control as a concrete and learnable skill. Similar to the second point, the observed pattern suggests a gradual design hypothesis that warrants experimental testing in future studies. Finally, because the program was delivered in a community setting through six 45–60-min sessions, it may have practical value for schools, after-school services, youth centers, or community-based digital wellness programs. However, scalability should not be assumed without further testing. Future implementations should include more systematic fidelity monitoring, attendance records, facilitator training checks, and participant engagement indicators to determine whether the program can be delivered consistently across different conditions.

### 4.4 Limitations and future research

Several limitations should guide interpretation. First, participants were not randomly assigned to conditions. Although quota-based allocation improved comparability in age, gender, and baseline Internet-use duration, unmeasured differences in motivation, family supervision, personality, prior concern about Internet use, or scheduling availability may have influenced the findings. Future studies should use randomized allocation, allocation concealment, and preregistered analyses where feasible. Second, the wait-list condition did not control for attention, expectancy, facilitator contact, or general self-monitoring. Active-control comparisons are needed to distinguish program-specific associations from these nonspecific influences. Third, DSE, DGI, and PIU were measured concurrently at posttest. The significant serial indirect association therefore does not establish that DSE changed before DGI or that DGI subsequently reduced PIU. The reverse and parallel models further showed that interpretation was specification-dependent, with DGI displaying the more consistent association with PIU. Multiple mediator assessments during and after the intervention are needed to evaluate temporal ordering. Fourth, all primary outcomes were self-reported. Because the program explicitly addressed PIU, self-regulation, and delay of gratification, it may have changed participants’ awareness of desirable responses or their interpretation of questionnaire items in addition to changing behavior. The short posttest interval and absence of assessor blinding may have amplified this differential response reactivity. Neither the decline in PIU in the wait-list group nor the significant Group × Time interactions can rule out this explanation. Without device-based measures or independent informant reports, the relative contributions of behavioral change and intervention-related reporting change cannot be determined. Future studies should incorporate screen-time records, app-use logs, ecological momentary assessment, and parent or teacher reports. Fifth, DGI dispersion increased markedly at posttest, especially in the wait-list group. Scoring and participant linkage checks did not identify an error, and robust change-score analyses supported a between-group difference; nevertheless, the source of the increased dispersion remains unexplained and warrants caution. Sixth, digital literacy itself was not measured. The study therefore cannot determine whether the program improved digital literacy per se or whether any such improvement accounted for the observed outcomes. The intervention should be understood as a multi-component digital literacy and digital wellbeing program that directly included self-control and delay-of-gratification activities. Seventh, the DGI measure assessed a generalized tendency across several life domains rather than digital-specific self-regulation. Future work should include measures tailored to digital reward contexts and evaluate discriminant validity among DSE, DGI, self-control, and PIU. Eighth, full attendance was not randomly determined; consequently, the per-protocol findings may reflect adherence-related selection and are descriptive only. Ninth, recruitment from one Chinese province through online and community channels limits generalizability, and participating families may have been particularly interested in digital wellbeing. Finally, only an immediate posttest was conducted, and the durability of the observed associations is unknown. Multi-site randomized studies with longer follow-up should also test moderators and experimentally compare different curriculum sequences.

## 5 Conclusion

This pretest-posttest quasi-experimental study found that participation in a 6-week multi-component digital literacy and digital wellbeing program was associated with more favorable self-reported changes in problematic Internet use, digital self-efficacy, and delay of gratification than a wait-list condition. The post-intervention data were statistically consistent with the hypothesized DSE → DGI → PIU indirect pathway, but alternative specifications indicated that DGI showed the more consistent association with PIU and did not establish temporal ordering. The findings are therefore preliminary and hypothesis-generating. Randomized, longitudinal, multi-method studies are needed to determine whether the observed differences reflect sustained behavioral change and to clarify the temporal relations among digital self-efficacy, delay of gratification, and problematic Internet use.

## Data Availability

The raw data supporting the conclusions of this article will be made available by the authors, without undue reservation.
